# Role identities of emergency medical services personnel and their associations with intention to leave the profession

**DOI:** 10.1186/s12873-024-01008-8

**Published:** 2024-06-05

**Authors:** Beeda Suokonautio, Anne Kouvonen, Hilla Nordquist

**Affiliations:** 1 Faculty of Social Sciences, University of Helsinki, Helsinki, 00014 Finland; 2 Centre for Public Health, Queen’s University Belfast, Belfast, BT12 6BA Northern Ireland; 3 South-Eastern Finland University of Applied Sciences, Kotka, 48220 Finland; 4 Faculty of Medicine, University of Helsinki, Helsinki, 00014 Finland

**Keywords:** Emergency medical services, Emergency medical services personnel, Paramedics, Role identity, Role conflict, Turnover

## Abstract

**Background:**

The scope of emergency medical services (EMS) has expanded from the urgent care of emergency patients to on-call healthcare services provided in the field with a holistic view of the patient’s wellbeing. This challenges EMS to find solutions to cover all demands, while simultaneously setting high skill requirements for EMS personnel. Understanding personnel is a critical element in developing functional and resistant EMS. The aim of this study was to investigate how Finnish EMS personnel emphasize the Emergency Medical Services Role Identity Scale aspects of caregiving, thrill-seeking, duty, and capacity; and if these role identities are associated with intention to leave the profession.

**Methods:**

We conducted a cross-sectional survey (*N* = 616, 52% women, mean age 32.9 years). Data were collected through social media platforms and analyzed with means, standard deviations, Mann-Whitney U-tests, Kruskal-Wallis H-tests, and binary logistic regression analyses.

**Results:**

Our results indicate that capacity is the most emphasized aspect among EMS personnel, and at the same time, it increases intention to leave EMS. Capacity was followed by caregiving, with no association with intention to leave. Duty and thrill-seeking were the least emphasized and were negatively associated with intention to leave. Additionally, there were also other factors that were associated with emphasizing EMS-RIS aspect and intention to leave.

**Conclusion:**

Capacity stands out most strongly in analysis being at the core of the role identity of EMS personnel and was associated with a higher likelihood of leaving intentions. Several other factors were also associated with the intention to leave. Future studies should examine the exact dimensions of capacity that are considered important among EMS personnel and why factors such as work experience are associated with intentions to leave.

**Supplementary Information:**

The online version contains supplementary material available at 10.1186/s12873-024-01008-8.

## Background

In recent years, the scope of emergency medical services (EMS) has expanded from the urgent care of emergency patients to on-call healthcare services provided in the field with a holistic view of the patient’s wellbeing. This trend can be seen across western countries, including Finland [1–4]. It has been influenced by the fast development of treatment methods, the increase of the educational level of professionals, and demographic changes, particularly rapid population aging. Consistent guidelines that comprehensively consider the healthcare system have been called for to develop better functioning services outside the hospital setting [5, 6]. At the same time, a clear definition of EMS and its sub-sections is seen as an essential factor for successful guidance [7, 8]. Many EMS professionals have worked through several stages of structural changes and are now working in a situation where the services, which were initially oriented towards acute care, is moving towards the provision of less urgent service. Today’s paramedics have acquired a considerable number of skills and competencies which are not yet fully utilized in EMS [9]. As a prerequisite for clarifying the structures of EMS, it has been suggested that the professional identity of the paramedic profession needs a more precise definition and analysis [10].

Donnelly et al. [11] have identified caregiving (“the caregiver role identity”), thrill-seeking (“seeking the excitement and stimulation of dramatic situations”), duty (“moral obligation”), and capacity (“confidence in ability in a variety of situations”) as key aspects of the professional role of paramedics. When looking at the literature from the caregiving point of view, paramedics have been found to build their professional identity strongly around the patient’s interest and treatment results, considering an honest relationship with the patient as one of the fundamental values, and experiencing personal responsibility for the patient’s care [12]. The professional ideal of equal and respectful care has also been presented as one of the core values of paramedics [13]. From the thrill-seeking point of view, the idea of a professional group that embodies some kind of heroism emerges. This is presented as a common external view of the field, whereas paramedics themselves often denigrate the term [14]. Mausz et al. [15] highlight a phenomenon where paramedics who identify with the thrill-seeking aspect talk about it in a “veiled manner”. The duty aspect can be seen as the willingness to provide public services combined with a certain amount of altruism [11, 15] and has been found to be more relatable to primary care paramedics than others [16]. From the perspective of the capacity aspect, paramedics feel that in emergency situations, they are expected to act regardless of their educational level or experience [17]. Independent decision-making and situation management [12], as well as the ability to make quick decisions [13] have been seen as essential parts of the work. In addition to these four aspects, two others have subsequently been identified: problem-solving (“a curiosity-driven and scientifically informed desire to solve problems”) and protecting (“actively protect people from harm”) [15].

The World Health Organization has published the Global Strategy on Human Resources for Health: Workforce 2030 [18]. It highlights the health workforce as a vital element of community resilience, and its training, deployment, retention, and performance as a worldwide cause for concern. Estimates show that the need for health workers will continue to increase for years to come. The strategy mentions turnover as one of the factors pushing estimate even higher. EMS plays an important role in the healthcare sector. Many researchers have investigated the factors which affect nurses’ intention to leave the profession, yet a deeper understanding of the *why* has yet to be attained and the mechanisms which lead to turnovers decoded [19]. The issue of role conflict between one’s personal expectations and actual work tasks has been associated with increased intention to leave [20]. Role conflict has been identified among paramedics and linked to a significant burden on employees` mental coping [15]. Such role conflict can expose the individual to work-related stress [21, 22]. In addition, it has been shown to affect task performance and organizational citizenship behavior [23] and to manifest itself as anger [15].

The aim of this study was to investigate how Finnish EMS personnel emphasize the Emergency Medical Services Role Identity Scale aspects of caregiving, thrill-seeking, duty, and capacity; and if these role identities are associated with intention to leave the profession. The research questions were:

1. How are the different EMS-RIS aspects emphasized among EMS personnel.
2. Are there statistically significant differences between demographic categories in terms of emphasizing the EMS-RIS aspects?
3. Which factors are associated with intention to leave the profession (i.e., EMS and the nursing sector completely).

## Methods

This research was carried out as a cross-sectional survey with paramedics and firefighters working in prehospital EMS in Finland as a target group. Persons participating only in first-response or voluntary responses, working only in transfer units, involved only in EMS physician units, or acting only as EMS field managers were excluded. Ethical approval for the study was received in spring 2021, and the data collection was carried out in summer 2021. The STROBE reporting guidelines were used (see Appendix 1).

### Setting

During the data gathering in 2021, the responsibility for organizing healthcare and social welfare, and fire and rescue services in Finland laid with hospital districts and municipalities. At that time, Finland was divided into five specific catchment areas, with each area overseeing its EMS. At the beginning of 2023, a healthcare and social welfare reform of the administrative structures transferred the organizing responsibility to 22 Wellbeing Services Counties.

The Finnish operative EMS system consists of units at different levels: first response units, basic level ambulances, advanced level ambulances, EMS field managers, ground-based EMS physician units, and helicopter EMS. In addition, there are many special units around the country (e.g., community paramedic units and tactical EMS units). Depending on the scenario and setting, the Emergency Response Center dispatches the most suitable unit(s) to the mission.

The Decree of Prehospital Emergency Care [24] has set the educational requirements for basic and advanced -level ambulances. At the basic level, there needs to be at least one person with a healthcare qualification with emergency medical orientation (minimum of practical nurse) in the ambulance. In advanced- level ambulances, there needs to be at least one person with a prehospital emergency care nursing qualification or a registered nurse with 30 ECTS of special emergency medical oriented additional training (bachelor’s level qualification). In both levels, the other crew member can be a firefighter or a healthcare professional. EMS physician units and EMS field managers work as support units to ambulances when needed, e.g., when an encountered patient requires demanding intensive care or in case of an accident with many casualties.

In Finland, paramedics assess the need for treatment and evaluate the most appropriate way to seek care. A patient will be transported to hospital if that is the most suitable option considering the patient’s state of health. Paramedics can independently start treatments and medications at the scene by following treatment instructions and their qualifications (basic/advanced level). When needed, paramedics can consult a physician for further instructions or ask a support unit to join the mission.

### Sampling and data collection

Participants were recruited through two closed Facebook groups with convenience sampling. Facebook groups were aimed at EMS professionals in Finland (“Ensihoidon uutiset”: The news from emergency care, and “Pelastuksen ja ensihoidon kahvihuone; foorumi uutisille ja keskusteluille”: The coffee room for fire service and emergency care; a forum for news and conversation). The inclusion criteria for these groups is that the person works in a fire service in EMS or is part of a stakeholder group. Some specific questions must be answered to get permission to join. A study recruitment ad and an open link to the survey on the Webropol 3.0 application were posted in these groups. Participation was voluntary, anonymous and based on informed consent. Data collection was carried out from June 9 to July 4, 2021 during the COVID-19 pandemic and the summer holiday season. A total of 616 responses were received.

### Variables

This study utilized a previously validated Emergency Medical Services Role Identity Scale (EMS-RIS) [11]. The EMS-RIS consists of 26 statements, with the scale from 1 to 7 (1 = entirely untrue, 7 = entirely true). It identifies four role identity aspects: caregiving, thrill-seeking, duty, and capacity. There are nine statements concerning caregiving (e.g., “*I enjoy learning about my patients´ lives”*, and “*Others tell me they believe I am empathic”*), six statements about thrill-seeking (e.g., “*I enjoy the rush of a good call”*, and ”*Like me, my co-workers enjoy the rush of a good call”*), seven statements about duty (e.g., “*The community I work in relies on me”*, and *“My family tells me they respect my dedication to public service”*), and four statements about capacity (e.g., “*I can be calm under pressure”*, and “*Others tell me they think I can be calm in difficult situations”*). The scale was translated from English to Finnish (first and last authors), and the translation was approved by two fully bilingual Finnish-English speakers (second author and an external person with a nursing background). In addition, the original English statements were also left visible for the participants to see. The development of the scale has been described elsewhere [11].

Background questions were based on the information from the literature review conducted for the study, as well as the background information generally used in EMS research. Gender (woman, man, or other), age (years), and years working in prehospital EMS (years) were asked. When investigating demographic differences in emphasizing EMS-RIS aspects, and factors associated with the intention to leave, gender was categorized as woman and man, as only two participants selected the option “other” for gender. Age was categorized into three groups: 18–29, 30–39 and 40 + years. Years of work experience in prehospital EMS were categorized as: ≤2, 3–6, 7–11 and ≥ 12.

The background variable describing the participant’s professional group was formed using questions concerning the level of education, the level of nursing or firefighter degree, and the task the person was performing in EMS at the time of the survey. The following five professional groups for background variables were formed: firefighter, practical nurse paramedic, registered nurse paramedic, emergency care nurse, and professional with a master’s degree.

The questionnaire included two questions concerning the intention to leave prehospital EMS work or the nursing sector completely: “I am seriously considering leaving prehospital EMS work during the next two years (moving to other positions in the nursing sector or only to firefighter tasks)” and “I am seriously considering leaving the nursing sector completely during the next two years” (leave this blank if you only have a firefighters’ degree). The answer options were “Yes”, “No”, and “Not sure”. In the Mann-Whitney U-test and binary logistic regression, the answers “No” and “Not sure” were merged.

From the questionnaire, only the EMS-RIS was obligatory, meaning that participants were able to leave background and intention to leave questions blank.

### Statistical analysis

The EMS-RIS had original values ranging from 1 to 7. The values were recoded from − 3 to + 3 following the example of Mausz et al. [16]. The characteristics of participants and analysis of EMS-RIS aspects are described as frequencies, percentages, means and standard deviations (SDs). The internal consistency of all EMS-RIS aspects was good. Cronbach’s alpha (α) for caregiving was 0.87 (α = 0.83; [16] and α = 0.90; [11]), for thrill seeking 0.82 (α = 0.84; [16], 2021 and α = 0.84; [11]), for duty 0.83 (α = 0.80; [16], 2021 and α = 0.84; [11]), and for capacity 0.83 (α = 0.87; [16] and α = 0.69; [11]).

A summary scale was formed for each EMS-RIS aspect (caregiving, thrill-seeking, duty, capacity) by adding the values together and then dividing the sum by the number of questions for each aspect. The highest score was defined as the participant´s dominant EMS-RIS aspect. As in a previous study [16], if there were multiple dominant aspects (i.e., the same score), the participant was excluded (*n* = 27). Dominant aspects are described with frequencies and percentages.

Non-parametric tests, the Mann-Whitney U-test and the Kruskal-Wallis H-test were used to determine if there were any statistical differences in emphasizing EMS-RIS aspects within each demographic category. The Mann-Whitney U-test was used to investigate gender differences and intention to leave prehospital EMS or the nursing sector completely. For categorical age, years of work experience in prehospital EMS, and professional group, the Kruskal-Wallis H-test was used, and pairwise comparisons command was applied to ascertain the exact groups between which statistically significant differences were found. These tests were conducted for a summary scale of each EMS-RIS aspect. Results are presented with statistical significance levels.

We ran a series of binary logistic regression analyses to examine whether gender, work experience in prehospital EMS, professional group, or EMS-RIS summary scales (caregiving, thrill-seeking, duty, capacity) were associated with the likelihood of intention to leave prehospital EMS work or the nursing sector completely. Intention to leave EMS and intention to leave the nursing sector completely were used as dependent variables where the answers “No” (intends to stay) and “Not sure” were merged and coded as 0 and “Yes” (intends to leave) was coded as 1. Work experience in prehospital EMS and all EMS-RIS summary scales were continuous variables. As only two participants selected the option “other” for gender, gender was coded as man (0) and woman (1). Emergency care nurses were used as the reference group when compared to other professional groups. Of the professional groups, firefighters and practical nurse paramedics were merged and coded as 1 due to the small number of participants in those groups; registered nurse paramedics were coded as 2; and professionals with a master’s degree were coded as 3. Logistic regression was performed using the forward selection (likelihood ratio) method. Results are presented as odds ratios (ORs) with their 95% confidence intervals (CIs).

All tests were performed with the significance level of 5% (*p* ≤ 0.05). The analyses were conducted between November 2022 to May 2023 using the IBM SPSS Statistics version 28. The results were reported during the year of 2023.

## Results

The characteristics of the participants and the mean values of the EMS-RIS aspects are shown in Table 1. Of the *n* = 599 participants who reported their gender, 51.9% were women and 48.1% men. The mean age was 32.9 years (SD 7.3), and the mean work experience was 8.1 years (SD 5.9). As Table 1 shows, nearly half of the participants were included in the professional group of prehospital emergency care nurses (45.3%). Of the participants, 17.7% intended to leave prehospital EMS work within two years, and 27.1% intended to leave the nursing sector completely (Table 1). Some participants had answered yes to both questions on intention to leave, and the total percentage intending to leave either prehospital EMS or the nursing sector completely was 33.1%.

**Table 1:** Characteristics and EMS-RIS aspects (mean scores) stratified by demographic category

Category	% within each category (*n*)	Caregiving mean (SD)	Thrill-seeking mean (SD)	Duty mean (SD)	Capacity mean (SD)
***All participants***	(616)	1.36 (0.83)	0.28 (1.17)	1.08 (1.00)	2.07 (0.81)
***Gender*** *(n = 599)*					
Women	51.9 (311)	1.51 (0.77)	0.39 (1.11)	1.16 (0.96)	2.02 (0.77)
Men	48.1 (288)	1.21 (0.85)	0.19 (1.21)	1.01 (1.02)	2.13 (0.80)
***Age group*** *(n = 483)*					
18-29y.	36.6 (177)	1.40 (0.84)	0.67 (1.05)	1.17 (1.00)	2.08 (0.82)
30-39y.	47.4 (229)	1.38 (0.75)	0.31 (1.12)	1.07 (0.94)	2.11 (0.71)
≥ 40y.	15.9 (77)	1.21 (1.23)	− 0.37 (1.27)	0.96 (1.23)	1.94 (1.19)
***Experience in EMS (years)*** *(n = 614)*					
≤ 2y.	15.1 (93)	1.40 (0.77)	0.61 (1.06)	1.33 (0.87)	1.98 (0.83)
3-6y.	30.9 (190)	1.31 (0.81)	0.45 (1.12)	1.08 (1.02)	2.03 (0.84)
7-11y.	30.6 (188)	1.40 (0.85)	0.27 (1.14)	1.02 (1.01)	2.12 (0.73)
≥ 12y.	23.3 (143)	1.35 (0.90)	− 0.13 (1.23)	0.99 (1.04)	2.09 (0.85)
***Professional group*** *(n = 598)*					
Firefighter	7.2 (43)	0.93 (0.89)	− 0.25 (1.09)	1.03 (1.08)	1.98 (1.00)
Practical nurse paramedic	16.7 (100)	1.38 (0.95)	0.35 (1.13)	1.19 (1.09)	2.04 (0.95)
Registered nurse paramedic	22.1 (132)	1.51 (0.66)	0.33 (1.18)	1.11 (0.96)	2.09 (0.63)
Emergency care nurse	45.3 (271)	1.32 (0.82)	0.29 (1.14)	1.03 (0.98)	2.04 (0.79)
Masters degree	8.7 (52)	1.47 (0.80)	0.57 (1.17)	1.18 (0.91)	2.30 (0.60)
***Intention to leave prehospital EMS*** *(n = 616)*					
Yes	17.7 (109)	1.29 (1.08)	− 0.15 (1.34)	0.66 (1.13)	2.02 (1.06)
No	65.6 (404)	1.38 (0.76)	0.40 (1.13)	1.27 (0.95)	2.09 (0.74)
Not sure	16.7 (103)	1.34 (0.80)	0.28 (1.02)	0.80 (0.87)	2.03 (0.76)
***Intention to leave nursing sector completely*** *(n = 591)*					
Yes	27.1 (160)	1.16 (1.11)	− 0.04 (1.29)	0.52 (1.15)	1.94 (1.11)
No	59.2 (350)	1.46 (0.72)	0.44 (1.12)	1.35 (0.85)	2.12 (0.68)
Not sure	13.7 (81)	1.42 (0.62)	0.35 (1.05)	1.01 (0.87)	2.07 (0.60)

The dominant EMS-RIS aspects by demographic category and intention to leave are displayed in Table 2. The capacity aspect had the highest mean scores in each demographic category (mean 2.07, SD (+/- 0.81) in all participants). Capacity was the dominant aspect for over half of the participants in every demographic category. The emphasis on capacity can be seen to get stronger among years of work experience (Tables 1 and 2). When comparing the categories of demographic variables, the only statistically significant difference appeared in gender: men had higher scores than women (*p* = 0.032).

The caregiving aspect had the second highest mean score (mean 1.36, SD (+/- 0.83) in all participants), followed by the duty aspect (mean 1.08, SD (+/- 1) in all participants). When comparing the mean values of the demographic categories, firefighters were the only group that emphasized duty over caregiving (Tables 1 and 2). This could also be seen when examining the differences in each categorical group. Firefighters had statistically significantly lower scores in the caregiving aspect compared to every other professional group. Also, gender (p = < 0.001) and intention to leave the nursing sector completely (*p* = 0.003) had statistically significant differences in experiencing caregiving, with men and those intending to leave having lower scores. When examining the differences in the duty aspect, intention to leave prehospital EMS or the nursing sector completely (p = < 0.001 in both) and work experience < 2 years compared to over 12 years (*p* = 0.04) stood out, with those with more work experience and those intending to leave having lower scores.

In terms of the dominant aspects, men, participants with a maximum of two years of work experience in prehospital EMS, practical nurse paramedics, emergency care nurses, and participants with no intention to leave prehospital EMS or the nursing sector completely were the groups in which duty was considered the second most dominant aspect (after capacity). In other groups, the second most dominant aspect was caregiving (Table 2).

The thrill-seeking aspect had the lowest mean score (mean 0.28, SD (+/- 1.17) in all participants). This aspect had the most variance among answers in each demographic category. Older participants and those with longer work experience emphasized this aspect less often. Firefighters were the only demographic category, in which nobody considered thrill-seeking as their dominant aspect (Tables 1 and 2).

All in all, participants intending to leave prehospital EMS or the nursing sector completely, as well as those who were aged 40 years or older, had lower mean scores in every EMS-RIS category.

**Table 2:** Dominant EMS-RIS aspects by demographic categories and intention to leave

Category	Caregiving % (*n*)	Thrill-seeking % (*n*)	Duty % (*n*)	Capacity % (*n*)
***All participants***	11.4 (70)	3.7 (23)	9.9 (61)	70.6 (435)
***Gender***				
Women (*n* = 311)	16.7 (52)	3.9 (12)	13.5 (42)	62.1 (193)
Men (*n* = 288)	5.6 (16)	3.8 (11)	5.9 (17)	79.9 (230)
***Age group***				
18-29y. (*n* = 177)	11.9 (21)	6.2 (11)	11.3 (20)	66.7 (118)
30-39y. (*n* = 229)	10.9 (25)	3.9 (9)	7.0 (16)	74.2 (170)
≥ 40y. (*n* = 77)	11.7 (9)	1.3 (1)	7.8 (6)	72.7 (56)
***Experience in prehospital EMS (years)***				
≤ 2y. (*n* = 93)	8.6 (8)	7.5 (7)	20.4 (19)	58.1 (54)
3-6y. (*n* = 190)	11.6 (22)	4.7 (9)	9.5 (18)	68.9 (131)
7-11y. (*n* = 188)	14.4 (27)	2.1 (4)	7.4 (14)	71.8 (135)
≥ 12y. (*n* = 143)	9.1 (13)	2.1 (3)	7.0 (10)	79.0 (113)
***Professional group***				
Firefighter (*n* = 43)	4.7 (2)	0	2.3 (1)	88.4 (38)
Practical nurse paramedic (*n* = 100)	11.0 (11)	4.0 (4)	13.0 (13)	65.0 (65)
Registered nurse paramedic (*n* = 132)	15.2 (20)	4.5 (6)	9.1 (12)	68.2 (90)
Emergency care nurse (*n* = 271)	10.0 (27)	3.3 (9)	10.3 (28)	71.6 (194)
Masters degree (*n* = 52)	13.5 (7)	7.7 (4)	9.6 (5)	69.2 (36)
***Intention to leave prehospital EMS***				
Yes (*n* = 109)	15.6 (17)	4.6 (5)	2.8 (3)	72.5 (79)
No (*n* = 404)	10.1 (41)	4.2 (17)	13.4 (54)	68.6 (277)
Not sure (*n* = 103)	11.7 (12)	1.0 (1)	3.9 (4)	76.7 (79)
***Intention to leave nursing sector***				
Yes (*n* = 160)	12.5 (20)	4.4 (7)	3.1 (5)	75.0 (120)
No (*n* = 350)	10.6 (37)	3.4 (12)	14.6 (51)	67.1 (235)
Not sure (*n* = 81)	14.8 (12)	4.9 (4)	6.2 (5)	70.4 (57)

Table 3 shows the associations of work experience, professional group and EMS-RIS aspects with intention to leave prehospital EMS. The likelihood of the participant intending to leave prehospital EMS seems to increase with years of work experience (OR 1.04; 95% CI 1.00-1.09) and with greater emphasis on the capacity aspect (OR 1.43; 95% CI 1.05–1.95). Moreover, participants with a master’s degree were more likely to have leaving intentions than emergency nurse paramedics (OR 5.09; 95% CI 2.42–10.73). A greater emphasis on the duty (OR 0.62; 95% CI 0.48–0.80) and thrill-seeking aspects (OR 0.74; 95% CI 0.58–0.93) were associated with a lower likelihood of intention to leave EMS (Table 3).

**Table 3:** Associations with intention to leave prehospital EMS

Statistically significant covariates in each Model	Model 1 OR (9% CI)	Model 2 OR (9% CI)	Model 3 OR (9% CI)	Model 4 OR (9% CI)	Model 5 OR (9% CI)
*Experience in prehospital EMS*	1.09 (1.05–1.12)	1.08 (1.04–1.12)	1.06 (1.02–1.10)	1.05 (1.01–1.09)	1.04 (1.00-1.09)
*Duty summary scale*		0.64 (0.51–0.79)	0.61 (0.48–0.76)	0.69 (0.54–0.88)	0.62 (0.48–0.80)
*Professional group*					
*Emergency nurse paramedic*			1.00 (ref.)	1.00 (ref.)	1.00 (ref.)
*Firefighter + practical nurse paramedic*			1.57 (0.88–2.80)	1.57 (0.87–2.82)	1.66 (0.92–2.99)
*Registered nurse paramedic*			1.02 (0.54–1.92)	1.06 (0.56.2.00)	1.07 (0.56–2.03)
*Masters degree*			4.3 (2.10–8.82)	5.09 (2.43–10.65)	5.09 (2.42–10.73)
*Thrill-seeking summary scale*				0.77 (0.61–0.97)	0.74 (0.58–0.93)
*Capacity summary scale*					1.43 (1.05–1.95)

In a similar way, longer work experience (1.06; 95% CI 1.02–1.10) and having a master’s degree (OR 2.06; 95% CI 1.04–4.09) were associated with a higher likelihood of intention to leave the nursing sector. A greater emphasis on the duty aspect (OR 0.47; 95% CI 0.38–0.59) decreased the likelihood of intention to leave the nursing sector (Table 4).

**Table 4:** Associations with intention to leave the nursing sector completely

Statistically significant covariates in each Model	Model 1 OR (9% CI)	Model 2 OR (9% CI)	Model 3 OR (9% CI)
*Duty summary scale*	0.47 (0.38–0.58)	0.48 (0.39–0.60)	0.47 (0.38–0.59)
*Experience in prehospital EMS*		1.06 (1.03–1.10)	1.06 (1.02–1.10)
*Professional group*			
*Emergency nurse paramedic*			1.00 (ref.)
*Firefighter + practical nurse paramedic*			0.60 (0.33–1.07)
*Registered nurse paramedic*			0.72 (0.42–1.22)
*Masters degree*			2.06 (1.04–4.09)

## Discussion

The aim of this study was to investigate how Finnish EMS personnel emphasize the EMS-RIS aspects of caregiving, thrill-seeking, duty, and capacity; and if these role identities are associated with intention to leave the profession (i.e., EMS and nursing sector completely). The results showed that capacity was the most emphasized aspect, followed by caregiving, duty, and thrill-seeking, in that order. Emphasis on the capacity aspect was associated with a higher likelihood of intention to leave EMS. Emphasis on the duty aspect was associated with a lower likelihood of intention to leave the nursing sector completely, whereas both duty and thrill-seeking were associated with a decreased likelihood of intention to leave EMS. Additionally, there were many demographic factors that were associated with emphasizing EMS-RIS aspects. Also, work experience and having a master’s degree were associated with a higher likelihood of intention to leave.

The capacity aspect was emphasized clearly over any other aspect, being also the most congruent EMS-RIS aspect according to participants’ views. As capacity can be defined as “confidence in ability in a variety of situations” [11], competence can be seen as a way to structure those situations at a practical level. Overall, previous studies have recognized that paramedics’ competence consists of many different areas, not just expertise in emergency care [25–27]. For example, Nilsson et al. [25] have categorized the required competencies for a paramedic under eight themes: Nursing Care, Value-based Nursing Care, Medical Technical Care, Care Environment Community, Care Environment Serious Events, Leadership Management, Supervision and Professional Conduct, and Research and Development. Moreover, the competency requirements for maintaining the ability to deal with urgent, life-threatening situations are vast, and the EMS´ shift toward non-urgent care expands the competency requirements even further [4, 27]. A greater emphasis on the capacity aspect was associated with an increased intention to leave EMS. The other aspects of role identity were not emphasized to the same extent as capacity. The caregiving aspect was the second most emphasized aspect overall. Firefighters, men, and those who intended to leave the nursing sector completely valued this aspect less than their peers. However, the caregiving aspect was not associated with intention to leave.

Our results showed that duty as the dominant aspect decreased notably after two years of work experience. The reasons for this could not be investigated in this study, but it might have something to do with an idealized view of the paramedic profession versus the reality after a few years of working in the field. In terms of the duty aspect, those who had no intention to leave prehospital EMS or the nursing sector completely valued this aspect somewhat more highly than those who were considering leaving. Feelings of personal responsibility and duty might be important reasons why one decides to stay in EMS. However, if duty is the thing that keeps you in the field, the work might become quite burdensome. Thrill-seeking was the least valued aspect. As discussed in the literature, heroism appears to be more part of the way an outsider might view EMS work [14]. That might be a problem when people with no experience in EMS apply to the field with a potentially inaccurate impression of what the work entails [28]. In this study, the duty and thrill-seeking aspects were positively associated with intention to stay in the profession; however, at the same time, they were the least emphasized aspects.

The finding that capacity, the most emphasized role identity aspect, was also associated with an increased intention to leave EMS is a phenomenon that needs some consideration. In a changing work environment, it should be clarified what tasks paramedics are trained for, what they think about their work roles, and what their requirements actually are now and possibly in the future [10, 29, 30]. In Finland it has recently been argued at the national level that the current training of emergency care nurses is not fully in line with the requirements of the field [24], which means that many things need to be learned at work. Paramedics themselves underline high quality target setting and clearly determined tasks, goals, and objectives as prerequisites for adequately running EMS [31]. Earlier studies have found that paramedics can feel that their competence has not been fully utilized due to the poor functionality of EMS [32]. In developing EMS, we should consider how to arrange the system so that personnel are able to utilize their whole capacity, meet organizational targets, and at the same time, maintain their work motivation. There are limits to the breadth of competencies one can absorb, maintain, and improve, especially with a sense of ability. Lack of challenge, and on the other hand, lack of perceived competence have been identified as two factors influencing novice nurses to leave the profession [33].

In a profession requiring high level competence, special knowledge, learning in the field, and a long training, labor shortages may become a huge problem as early as in the near future. In this study, a third of the participants were considering leaving the profession (either prehospital EMS or the nursing sector completely), thus it can be argued that further research on finding ways to support employees to stay would be important. Years of experience in prehospital EMS was not associated with intention to leave, so it can be assumed that leaving happens at all experience levels. It is notable that, in this study, emergency care nurses with four years of bachelor’s level education were less likely to intend to leave prehospital EMS, but more likely to intend to leave the nursing sector completely. This might be due to the overall professional identity, which may be oriented toward out-of-hospital EMS and the move to other health sectors might not be seen as inviting. Moreover, participants with a master’s degree were more likely to consider leaving prehospital EMS and the nursing sector completely. There are many competencies that could be utilized in EMS if more thought were given to career paths in this sector [34]. One might not be motivated to continue doing the exact same tasks after obtaining a postgraduate degree. In addition, a high-quality mentoring system could be seen as an important factor in supporting newcomers. In an earlier study, paramedics highlighted the positive effect of “an experienced and competent colleague”, which contributed to their feelings of security in the profession and increased job satisfaction [32]. Therefore, professionals with greater experience play an important role in supporting colleagues, sharing knowledge, having hands-on experience and tacit knowledge.

## Limitations of the study

This study had some limitations. First, self-selection bias is inherent with convenience sampling. Those with intention to leave might have been more motivated to participate, since this type of study could be seen as more appealing to them. Second, the mean age of the participants was relatively low. However, due to the acute nature of emergency care and physically challenging duties, this age distribution might be quite accurate in the paramedic profession. There are no national statistics concerning the age distribution of paramedics. On the other hand, 133 participants did not want to state their age, so the actual mean age of the participants may be higher. Third, the data collection was conducted during the COVID-19 pandemic and the summer holiday season in 2021. The pandemic has been studied as an overall burdensome period in healthcare [35, 36] and thus the timing may have increased the leaving intentions. In Finland, the summer holiday season spans from May to September, and in EMS, all summer holidays are staggered due to the 24/7/365 operation. Following Facebook groups presumably occurs more during leisure time than work hours and does not necessarily pause during holidays. Therefore, the organization of data collection during the holiday season may not have impacted the results but it is important to acknowledge when interpreting the findings. Fourth, the EMS-RIS was developed in a North American EMS environment which may in some parts differ from the Finnish context. However, before releasing the survey, it was piloted with a test group, and minor adjustments were made according to their feedback. In this way, the technical functionality of the questionnaire was also ensured.

This study also had a number of strengths. The recruitment method via social media platforms enabled us to reach a large number of potential participants across the country. It has been argued that this type of recruitment method is the most successful among nursing and healthcare providers [37]. In the questionnaire, participants were asked in which region they worked, and the study was nationally comprehensive.

A total of 616 responses were received. According to Venesoja et al., Finland had 3,898 full-time EMS workers in 2019 [38]. Based on this, the total response rate of the study was 16%. However, there are no separate public statistics on professionals employed in prehospital EMS, which is a limitation for defining the target population size. In addition, firefighters were also included in this study, and their response rate was low, as only 43 participated. Based on the number of times the questionnaire forms were opened (1,027 times) and responses returned, the completion rate was 60% [37], which can be considered high.

The results of the study are generalizable to the Finnish paramedic profession, and also at least indicative for other countries where paramedics have a similar job description and educational background as in Finland.

## Conclusion

Capacity was the most emphasized aspect of the EMS-RIS scale among EMS professionals and at the same time it increased the likelihood of intention to leave EMS. In addition, there are several factors that were associated with different EMS-RIS aspects and intention to leave.

In order to better understand the reasons behind intentions to leave EMS, we need a deeper understanding of the topic. For example, it should be studied more carefully which aspects of capacity are considered important among EMS personnel, how they can utilize their capacity in practice, and how those aspects are associated with intentions to leave. It should also be studied why factors such as work experience are connected to leaving intentions. That kind of knowledge could be utilized in decision-making when developing EMS structures.

### Electronic supplementary material

Below is the link to the electronic supplementary material.

Supplementary Material 1

## Data Availability

The datasets generated and analyzed during the current study are not publicly available due to the data handling described in the research notice to the participants, but are available from the corresponding author on reasonable request and respecting the data usage rights promised to the participants as well as their secure storage.

## Abbreviations

EMS: Emergency medical services
EMS-RIS: Emergency Medical Services Role Identity Scale
