# Supplementary material for: Role identities of emergency medical services personnel and their associations with intention to leave the profession
Source: BMC Emerg Med. 2024 Jun 5;24:96. doi: 10.1186/s12873-024-01008-8 (PMC11155154; doi:10.1186/s12873-024-01008-8)
Supplement: Supplementary file 1 — Supplementary Material 1 [file 12873_2024_1008_MOESM1_ESM.docx]

**APPENDIX 1:** STROBE Statement – Checklist of items that should be included in reports of cross-sectional studies

|  | **Item**  **No** | **Recommendations** | **Reported** |
| --- | --- | --- | --- |
| **Title and abstract** | 1 | (a) Indicate the study’s design with a commonly used term in the title or the abstract | Abstract |
|  |  | (b) Provide in the abstract an informative and balanced summary of what was done and what was found | Abstract |
| **Introduction** | | | |
| Background/rationale | 2 | Explain the scientific background and rationale for the investigation being reported | Background |
| Objectives | 3 | State specific objectives, including any prespecified hypotheses | Background |
| **Methods** | | | |
| Study design | 4 | Present key elements of study design early in the paper | Methods |
| Setting | 5 | Describe the setting, locations, and relevant dates, including periods of recruitment, exposure, follow-up, and data collection | Methods |
| Participants | 6 | (a) Give the eligibility criteria, and the sources and methods of selection of participants | Methods |
| Variables | 7 | Clearly define all outcomes, exposures, predictors, potential confounders, and effect modifiers. Give diagnostic criteria, if applicable | Methods |
| Data sources/ measurement | 8 | For each variable of interest, give sources of data and details of methods of assessment (measurement). Describe comparability of assessment methods if there is more than one group | Methods |
| Bias | 9 | Describe any efforts to address potential sources of bias | Discussion |
| Study size | 10 | Explain how the study size was arrived at | Methods, based on received responses |
| Quantitative variables | 11 | Explain how quantitative variables were handled in the analyses. If applicable, describe which groupings were chosen and why | Methods |
| Statistical methods | 12 | (a) Describe all statistical methods, including those used to control for confounding | Methods |
|  |  | (b) Describe any methods used to examine subgroups and interactions | Methods |
|  |  | (c) Explain how missing data were addressed | Methods, Discussion |
|  |  | (d) If applicable, describe analytical methods taking account of sampling strategy | Limitations |
|  |  | (e) Describe any sensitivity analyses | Methods |
| **Results** | | | |
| Participants | 13 | (a) Report numbers of individuals at each stage of study—eg numbers potentially eligible, examined for eligibility, confirmed eligible, included in the study, completing follow-up, and analysed | Results, Table 1 |
|  |  | (b) Give reasons for non-participation at each stage | Methods, Discussion |
|  |  | (c) Consider use of a flow diagram | Not used |
| Descriptive data | 14 | (a) Give characteristics of study participants (eg demographic, clinical, social) and information on exposures and potential confounders | Results, Tables 1-2 |
|  |  | (b) Indicate number of participants with missing data for each variable of interest | Results, Tables 1-2 |
| Outcome data | 15 | Report numbers of outcome events or summary measures | Results, Tables 1-2 |
| Main results | 16 | (a) Give unadjusted estimates and, if applicable, confounder-adjusted estimates and their precision (eg, 95% confidence interval). Make clear which confounders were adjusted for and why they were included | Results, Tables 3-4 |
|  |  | (b) Report category boundaries when continuous variables were categorized | Methods, Results: Tables 1-2 |
|  |  | (c) If relevant, consider translating estimates of relative risk into absolute risk for a meaningful time period | Not Applicable |
| Other analyses | 17 | Report other analyses done—eg analyses of subgroups and interactions, and sensitivity analyses | Results, Table 1-4 |
| **Discussion** | | | |
| Key results | 18 | Summarise key results with reference to study objectives | Discussion |
| Limitations | 19 | Discuss limitations of the study, taking into account sources of potential bias or imprecision. Discuss both direction and magnitude of any potential bias | Discussion |
| Interpretation | 20 | Give a cautious overall interpretation of results considering objectives, limitations, multiplicity of analyses, results from similar studies, and other relevant evidence | Discussion |
| Generalisability | 21 | Discuss the generalisability (external validity) of the study results | Discussion |
| **Other information** | | | |
| Funding | 22 | Give the source of funding and the role of the funders for the present study and, if applicable, for the original study on which the present article is based | Footnotes |

Equator network. SROBE Statement-Checklist of items that should be included in reports of cross-sectional studies. 2023. https://www.equator-network.org/. Accessed 1 Jan 2023.
